# Photoacoustic image patterns of breast carcinoma and comparisons with Magnetic Resonance Imaging and vascular stained histopathology

**DOI:** 10.1038/srep11778

**Published:** 2015-07-10

**Authors:** M. Heijblom, D. Piras, M. Brinkhuis, J. C. G. van Hespen, F. M. van den Engh, M. van der Schaaf, J. M. Klaase, T. G. van Leeuwen, W. Steenbergen, S. Manohar

**Affiliations:** 1 Biomedical Photonic Imaging, MIRA Institute for Biomedical Technology and Technical Medicine, University of Twente, P.O. Box 217, 7500 AE, Enschede, The Netherlands; 2 Center for Breast Care, Medisch Spectrum Twente, P.O. Box 50.000, 7500 KA, Enschede, The Netherlands; 3 Laboratory for Pathology East Netherlands, P.O. Box 516, 7550 AM, Hengelo, The Netherlands; 4 Biomedical Engineering and Physics, Academic Medical Center, University of Amsterdam, P.O Box 22700, 1100 DE, Amsterdam, The Netherlands

## Abstract

Photoacoustic (optoacoustic) imaging can visualize vasculature deep in tissue using the high contrast of hemoglobin to light, with the high-resolution possible with ultrasound detection. Since angiogenesis, one of the hallmarks of cancer, leads to increased vascularity, photoacoustics holds promise in imaging breast cancer as shown in proof-of-principle studies. Here for the first time, we investigate if there are specific photoacoustic appearances of breast malignancies which can be related to the tumor vascularity, using an upgraded research imaging system, the Twente Photoacoustic Mammoscope. In addition to comparisons with x-ray and ultrasound images, in subsets of cases the photoacoustic images were compared with MR images, and with vascular staining in histopathology. We were able to identify lesions in suspect breasts at the expected locations in 28 of 29 cases. We discovered generally three types of photoacoustic appearances reminiscent of contrast enhancement types reported in MR imaging of breast malignancies, and first insights were gained into the relationship with tumor vascularity.

In women, breast cancer is the most frequently occurring malignancy and the leading cause of cancer death^1^,^2^. In 2008, about 1.4 million women were diagnosed with breast cancer, which accounts for 23% of all new cancer cases^1^. Besides, in the same year, with 0.5 million deaths, breast cancer was the cause of 14% of all female cancer deaths. In future, it is expected that while the high prevalence in the affluent world regions stabilizes, incidence rates and mortality will strongly rise in the low and medium Human Development Index (HDI) regions.

Advances in fundamental breast cancer research with steady translation in adjuvant chemotherapy as well as radiation, hormonal, and targeted therapies on the one hand, and introduction of screening programs on the other, have lead to progressively decreasing death rates^3^. Imaging plays a crucial role across the entire breast cancer care spectrum for detection, diagnosis, neoadjuvant therapy monitoring, guiding probes for biopsies, guiding interventions and for surveillance^4^,^5^. Despite substantial progress with ever-improving spatial resolutions, the current crop of breast imaging methods still falls short of requirements^5^. X-ray mammography (XRM), the only technique for screening and the mainstay of diagnosis^4^, has a low positive predictive value (PPV) leading to unnecessary secondary investigations including biopsies^6^. It also suffers from other well-known drawbacks such as reduced accuracy in breasts rich in radiodense glandular tissue where the sensitivity falls from around 90% to between 40–60%^7^, the use of ionizing radiation (albeit at low doses), and the requirement for painful breast compression. Ultrasound (US) imaging remains an adjunct to XRM with value in diagnostic assessment^4^ applied predominantly for differentiation between solid masses and cysts, for palpable mass evaluation where XRM fails or is not recommended, and for guiding biopsies. It provides high negative predictive value (NPV) but at the expense of a high false positive rate, and has the limitation of being operator dependent and not sufficiently standardized^8^. A major reason for the limitations of both XRM and US imaging is that they report on anatomic and morphological differences between the tumor (or sometimes associated calcifications) and healthy tissue, which can remain subtle in many cases especially in early disease.

One of the integral hallmarks of cancer has been proposed to be angiogenesis, the production of new blood vessels, induced surprisingly early to support malignant and even pre-malignant phases in the multistage development of invasive cancers^9^. This process results in a locally increased microvascular density (MVD) with abnormal vessels which are dilated and tortuous. Tumor vasculature is studied in pathology using microscopy on immunohistochemically (IHC) stained tissue specimens^10^, with microvessel quantitation being a prognostic parameter^11^.

The significance of tumor angiogenesis has been recognized beyond the fields of histopathology, being also appreciated among others in the domain of radiology. Pre-operative Dynamic Contrast Enhanced Magnetic Resonance Imaging (MRI), which tracks the passage and kinetics of contrast agents through the vasculature, exploits the altered MVD and abnormal vessel characteristics for sensitive tumor identification^12^,^13^. MRI is gaining a niche role in problem solving where XRM and US return uncertain findings, for surveillance when high familial risk is present, where cancer recurrence is suspected in fibrotic breasts, and for evaluation of multifocii and contralateral lesions^14^. The method does not employ ionizing radiation and has good spatial resolution. However, MRI requires contrast agents, its quality depends on the patient’s hormonal status^12^, and there exist considerable exceptions and overlaps in contrast agent uptake and kinetics between malignancies and benign tumors, leading to poor specificity^15^,^16^. Besides, poor PPV (though better than in XRM), high expense and consequent inaccessibility, are major drawbacks for the use of MRI. Despite these limitations, the high sensitivity that is reported to be between 89–100% in breast cancer detection for MRI^12^,^13^, strongly suggests that tumor vascularization is a reliable imaging target.

Light can be used to visualize breast cancer due to the substantial and specific optical absorption possessed by hemoglobin (Hb) and its oxygenated variant (HbO) in tumor vascularization^17^. Near-infrared light has the advantage of being non-ionizing and uses relatively simple and low-cost instrumentation. However, while near-infrared light can penetrate deep in tissue, it undergoes multiple scattering, which results in poor spatial resolution. This causes a smearing out of the contrast and thus reduces the contrast-noise ratio (CNR), adversely affecting the detectability of small cancers and those at early stages of progression. Further, spatial averaging causes loss of information regarding the heterogeneous vascular distribution in the cancer, which can be a further handle in discrimination between malignant and benign lesions, as used in MRI^12^,^13^.

Photoacoustic (also called optoacoustic) imaging is a relatively new imaging technique that can produce high resolution 3D images of optical absorption distributions in tissue and is unaffected by light scattering^18^,^19^. The method is based on the conversion of absorbed pulsed light energy into acoustic energy via thermoelastic expansion at sites where light absorption has taken place. Acoustic transients with frequencies in the ultrasound regime travel with low and known velocities through tissue. These acoustic waves are scattered and attenuated considerably less in their propagation than light. Detection using US transducers permits a reconstruction of the spatial distribution of the acoustic sources with resolutions dictated almost solely by the bandwidth and spatial sampling of the detectors used^18^.

The application of near-infrared (NIR) PA for breast imaging was first suggested by Oraevsky *et al.*^20^ and Kruger & Liu^21^. The group of Oraevsky showed first patient results using the laser-based optoacoustic imaging system (LOIS) in 2001^22^. Using the Twente Photoacoustic Mammoscope (PAM), a lab-made prototype PA breast imager, we demonstrated in small patient cohorts of 5 and 10 patients^23^,^24^ that the method is able to visualize malignancies with high imaging contrast. Interestingly, the contrast appeared to be independent of the mammographically estimated breast density^24^. In these studies, a wavelength of 1064 nm was used for excitation, and a planar ultrasound detector array with 590 elements was used for detection^25^. In this version, parallel acquisition of detection elements was not possible with the consequence that within the measurement time of 25 minutes, a field-of-view (FOV) of the breast surface of only 45 × 45 mm could be achieved^23^,^24^.

While the patient imaging results have been exciting in demonstrating technical feasibility of various embodiments of PA breast imagers^23^,^24^,^26^,^27^,^28^,^29^, largely case studies have been performed. In the current work, for the first time the approach is question-driven: what are PA appearances of malignancies? Can these be related to tumor vasculature? The knowledge of the relation between PA appearance and tumor vascularization can potentially contribute to the development of PA image descriptors which in the long run may serve as diagnostic criteria for the technique. We used an upgraded version of the instrument, where parallel acquisition of 10 elements at a time is enabled to permit imaging of a FOV of 90 × 85 mm in 10 minutes. This larger FOV allows us to include a wider variety of lesions and lesion sizes. A larger FOV is also required to obtain higher fidelity in size and shape reconstruction, and allow more accurate visualizations of the PA appearances. In addition to comparisons with XRM and US imaging, for the first time we compare the PA images with MR images and vascular staining in histopathology. The results give us new insights into PA lesion appearances, and the relationship between these appearances and tumor vascularity.

## Results

### General findings

Between April 2012 and May 2013, the upgraded PAM was used to image suspect and highly suspect breasts of 29 patients. The results of those 29 patient measurements gave rise to a research question which is further investigated in the 14 patients presented in this manuscript. In contrast to previously reported clinical data^23^,^24^,^26^,^27^, lesions presented not only as homogenous masses, but exhibited various types of appearances. We find these patterns of PA intensity somewhat reminiscent of the contrast enhancement types generally reported in MRI of breast malignancies^30^, and we adopted the same descriptions as in the MR nomenclature. We identified generally the following PA image patterns:

1. Mass-like appearance consisting of a confined region of high intensity, which may be either homogenous or heterogeneous in spatial distribution, and regular or irregular in shape;
2. Ring appearance consisting of a region of high intensity completely or partially surrounding an area with lower intensity;
3. Non-mass or scattered appearance comprising scattered foci of high intensity within an extended region.

To investigate the relation between PA appearance pattern and tumor vascularization, for each appearance type, one or more representative tumors were selected for CD31 immunohistochemistry (IHC) on tissue sections to visualize the density and distribution of vascularity. In total, 6 of such cases were included for the CD31 staining. From these 6 patients, pre-operative MR images were available for 3 cases and for 8 additional patients not included in the CD31 histopathology protocol, MR images were also available because this was being performed for medical purposes. Here the results for these 14 patients are described in detail, in the first section PA results are compared to the available MRI results (11 cases) and in the subsequent section PA results are compared to the available CD31 immunohistochemistry results (6 cases). For all described cases XRM and US images were made.

### Comparison between PA images and MR images (11 cases)

**Figure 1:**
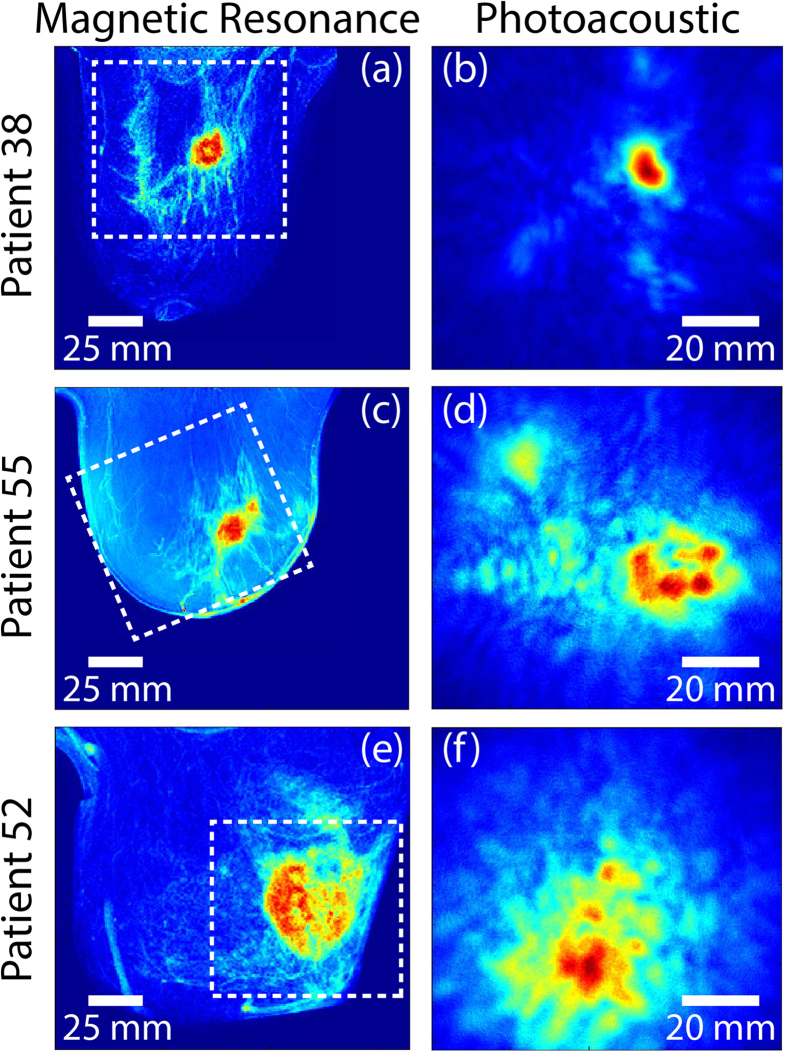
MR (left) and PA (right) cranio-caudal (CC) average intensity projections (AIP) of the breast for three cases. **(a–b)** Patient P38, having an IDC, grade 3 of 19 mm; **(c–d)** Patient P55, having an IDC, grade 2 of 34 mm; **(e–f)** Patient P52, having an IDC (unknown grade) of more than 50 mm. The dashed box in the MR image indicates the area from which the PA image is acquired.

Figure 1 shows average intensity projections (AIPs) in cranio-caudal (CC) direction of MR (left) and PA (right) reconstructed data for three cases with increasing histopathological size. The dashed box in the MR image indicates the region where the PA FOV has been acquired. The first two cases P38 and P55 (Fig. 1(a) and Fig. 1(c) respectively) are representative for lesion sizes up to 35 mm in conventional XRM. There is good correspondence in lesion co-localization in the PAM and MR images, and remarkably also in gross lesion appearance and shape. For such small to medium sized lesions, similar observations are made in 7 of the *n* = 8 cases regarding co-localization (Table 1), and in all *n* = 8 cases regarding appearance and shape. In the one case of unsatisfactory co-localization (P56), the breast was severely tilted during the PA measurement, making it difficult to exactly orient the PA image with respect to the MR (and XRM) images. However, also in this case, the lesion position in the PA image matched well with the expected mass position based on palpation and US imaging.

In the third case P52 (Fig. 1e,f), lesion co-localization between MR and PA images is good, while the resemblance in shape and appearance is minor. Similar observations are made in the 2 additional cases (Table 1) where the histopathological lesion size is >40 mm (in case histopathology was not available, the size on x-ray imaging was taken as gold standard). In all the *n* = 3 (P40, P49 and P52) cases lesions presented as non-mass with a scattered appearance in the PA images, as can also be seen in the lower rows of Table 1. It is likely that the lesions are too large relative to the FOV to have an appropriate PA reconstruction of the complete malignancy and its surroundings.

The estimated lesion sizes are significantly larger in MR images than in PA images for a number of cases. This discrepancy is mostly the consequence of the presence of spiculae in the MR images, which are not visible in PA images. In these cases (P55, P58, P70), the estimated lesion sizes in PA images were closer to the histopathologically defined lesion size (Table 2).

### Comparison between PA appearance and histopathology (6 cases)

**Figure 2:**
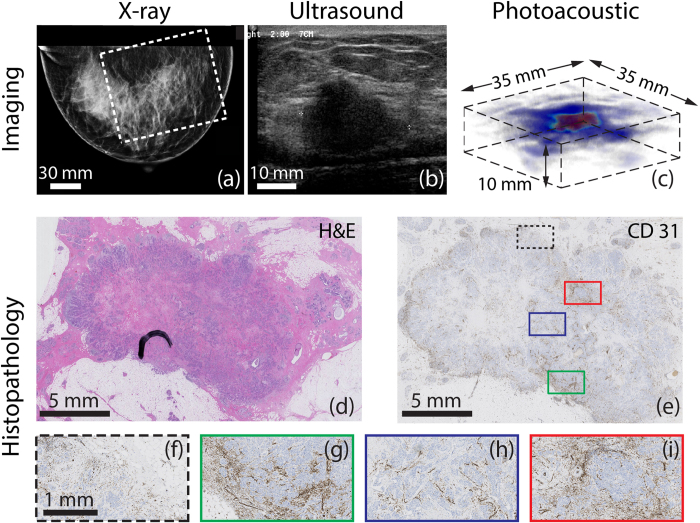
Example of photoacoustic mass-like appearance seen in 44 year old patient (P38, Case 1) with infiltrating ductal carcinoma (IDC). **(a)** The cranio-caudal x-ray mammogram of the right breast does not show any abnormalities and is judged as BI-RADS 1. The white box indicates the field-of-view (FOV) of the PA image. **(b)** Ultrasound image of palpable mass, reveals 22 mm highly suspect mass. (For MR image see Fig. 1(a)) **(c)** A volume rendering of part of the PA data reveals confined high intensity area, which we define as mass appearance. The corresponding CC AIP is seen in Fig. 1(b) **(d)** Post-surgical histopathological investigation reveals 19 mm grade 3 IDC, which shows **(e)** in the CD31 stained tumor slide a hetereogeneous distribution of vascularity. This is further highlighted in the four selected areas **(f)–(i)** at the margins and in the center of the tumor.

**Figure 3:**
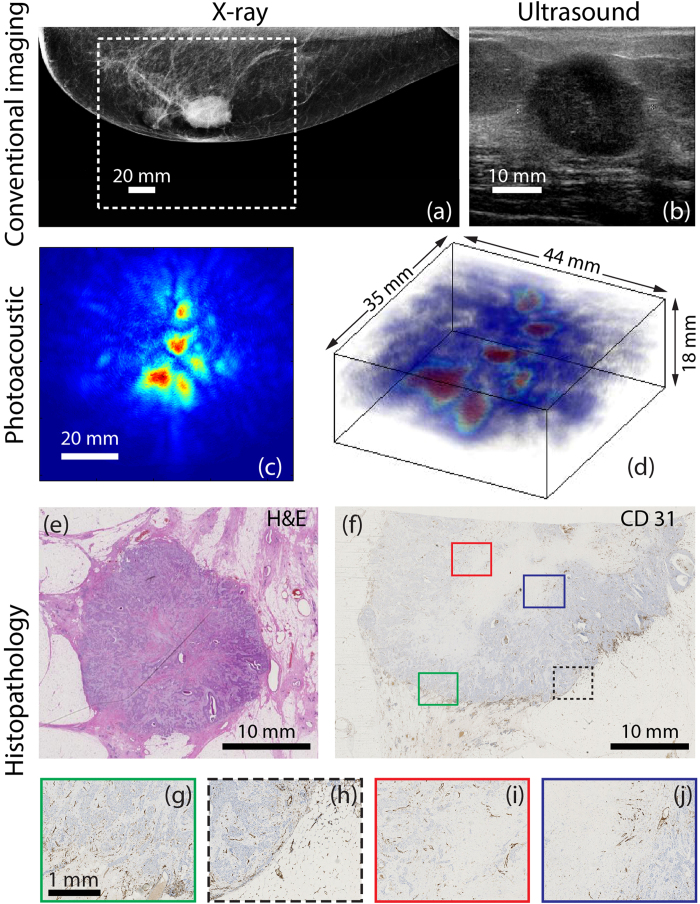
Example of photoacoustic ring-like appearance observed in 69 year old (P47, Case 2) with IDC **(a)** The CC x-ray mammogram of the left breast shows a highly suspect mass measuring about 24 mm in diameter. The white box indicates the FOV of PA image. **(b)** Ultrasound image of the palpable mass reveals highly suspect mass measuring 29 mm. **(c)** MIP in CC view of PA volume presents as a collection of high intensity zones surrounding an area of low intensity. **(d)** A volume rendering of PA data reveals the ring appearance. **(e)** The whole mount H&E stained slide reveals IDC of 24 mm in diameter, associated with central fibrosis. **(f)** The CD31 section shows relatively low vascularity, mostly pronounced at the lesion borders. At the center a large area without vascularity can be observed. **(g)–(j)** Details at the border and center of the malignancy showing higher vascularity at border regions and an avascular center.

**Figure 4:**
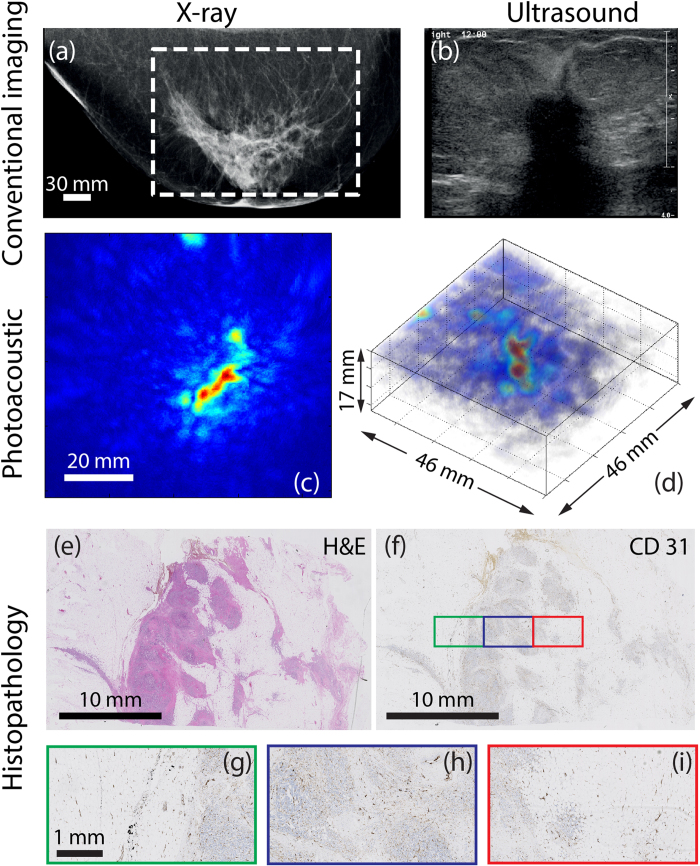
Example of photoacoustic non-mass-like appearance in 67 year old (P39, Case 3) with IDC. **(a)** The highly suspect CC x-ray mammogram of the right breast shows a 5 cm architectural distortion and nipple retraction. The white box indicates the FOV of PA image. **(b)** Ultrasound image reveals irregular shaped highly suspect hypoechoic mass (at least 5 cm) with hyperechoic border, partly invading into the subcutis. **(c)** PA MIP shows lesion as discrete regions of moderate intensity, defined as non-mass appearance, with the abnormal zone roughly 41 mm along the longest axis. **(d)** A 3D volume of the suspicious area shows the difficulty in assigning such a lesion. **(e)** The H&E section through part of the tumor in sagittal direction, reveals the grade 3 IDC, where tumor cells are organized in non-cohesive fields within fibrous stroma. Combining several such slices, the total malignancy measures 63 mm. **(f)** The CD31 section and details in **(g)-(i)** shows relatively low vascularity, mostly confined in the tumor nests and less prominent in the sclerotic stroma.

In general, the sizes and shapes of lesions in PA images showed similarities with the histopathologically derived sizes and shapes of the lesions. Further in 5 of the 6 cases of the CD31 stained slices, there was good correspondence between PA lesion appearances and the microvascularity patterns. Specifically, the following could be said:

- For all three cases of mass appearance in PA images (P38, P55 and P70), the microvascularity was spread over the entire lesion (Table 2 and Fig. 2).
- For both cases of ring appearance in the PA images (P47 and P37), the histopathology report described tumor nests confined to the lesion border, surrounding a fibrotic core. However, only in the case of P47, the CD31 stains showed the expected higher vascularity at the lesion border (Table 2 and Fig. 3). In the case of P37 the vascularity (See Case 4) was observed throughout the lesion.
- For the case of the non-mass appearance (P39), the microvascularity was pronounced in fields of tumor cells non-cohesively distributed over the entire lesion (Table 2 and Fig. 4).

We now present detailed results of four specific cases showing the three PA appearance types with comparisons with MR images (if available) and histopathology.

### Case 1 (P38) – PA mass appearance for IDC

The 44 year old Caucasian patient was under annual surveillance following a history of IDC in the left breast. No abnormalities were observed in the XRM of both breasts. However, the presence of a palpable mass in the right breast prompted a localized US investigation. Figure 2a shows the mammogram of the suspect right breast which was designated as BI-RADS 1 since no irregularities could be perceived. Figure 2b shows the US image, which reveals the presence of an unsharply delineated, irregular, hypoechoic mass of 22 mm which is highly suspect for malignancy (BI-RADS 5). Histopathological investigation of the tissue specimen after core needle biopsy (CNB), revealed IDC. Because of the discrepancy between XRM and US, a preoperative MRI was performed. The average intensity projection (AIP) of the MR volume is shown in Fig. 1a, where the malignancy is clearly identified as a 22 mm heterogeneous intensity distribution, showing some ring enhancement at the expected lesion location. Enhancement of the surrounding parenchymal tissue is also seen.

The reconstructed PA volume is shown as an AIP in CC direction in Fig. 1b, and as a direct volume rendering in Fig. 2c. The PA FOV is indicated by the dashed box in the MR and XRM images (Fig. 1a and Fig. 2a respectively). The lesion measures 19 mm in the AIP and is defined as being a ‘mass’ because of the confined region of high intensity. The contrast of the lesion with respect to background (*C*) is estimated to be 5.1. There is good correspondence between PA and MR images with respect to location, size and shape of the lesion. The ring enhancement of MRI is not visible in the PA image volume.

The H&E stained slide (Fig. 2d), used for normal histopathological diagnosis, indicates the presence of a 19 mm grade 3 IDC. Additional immunohistochemical (IHC) staining (not shown) revealed the triple negative hormonal status (ER-, PR- and HER2/neu-) of the lesion. The CD31 stain (brown) indicates the presence of endothelial cells which line blood vessels. The staining and thus the vascularity is spread over the tumor (Fig. 2e and details in Fig. 2f–i), although in certain areas the staining appears more pronounced at the lesion border. Both in the center (red and blue inset) and the periphery (black and green inset) of the lesion, fields with higher or lower vascularity are observed which correlates with the heterogeneity observed in the MR images. However, the ring appearance that was visible in MR images cannot be recognized from this particular tumor slide.

In the other two patients with mass appearance in the PA images, the vascularity was spread over the lesion. These two lesions also presented as mass in the post-contrast MR images.

### Case 2 (P47) – PA ring appearance for IDC

The 69 year old Caucasian patient presented with a highly suspicious palpable mass in her left breast. Figure 3a shows the CC x-ray image of the left breast, which reveals a 24 mm lesion central in the breast highly suspect because of its inhomogeneity and indistinct margins and the associated skin thickening. The US image (Fig. 3b), shows an irregularly shaped mass with an inhomogeneous internal echo structure and partly indistinct margins.

The MIP of the reconstructed PA volume (Fig. 3c) shows high intensity regions distributed around an area with low intensity. The ensemble of PA hotspots co-localize well with the mass on the x-ray image (see dashed box in XRM image marking PA FOV). The contrast of this ensemble (*C*) with respect to background is estimated to be 5.5. We describe the PA presentation of the lesion as ring appearance; the ring measures 24 mm in diameter on the MIP. This appearance may be better appreciated in the volume visualization of the PA reconstruction (Fig. 3d).

The H&E section through the center of the lesion (Fig. 3e) reveals a 24 mm grade 2 IDC. The tumor core is cell-poor and connective tissue rich, evident from the absence of stained nuclei, in contrast to the lesion borders which are purple due to nuclear staining. This is consistent with central fibrosis in the tumor. Figure 3f shows the CD31 section of part of the tumor including both central and margin areas. Although the vascularity is not pronounced, it appears to be confined to the tumor borders (Fig. 3g–h and correspondingly colored boxes in Fig. 3f) primarily at the interface between the tumor and normal tissue. At the center, low vascularity is observed (Fig. 3i–j and corresponding boxes in Fig. 3f). This appearance of fields of vascularity surrounding an avascular area corresponds with the PA lesion ring appearance of high intensity zones surrounding an area with low intensity.

### Case 3 (P39) – PA non-mass appearance for IDC

Clinical investigation of the suspicious right breast of the 67 year old Caucasian following an abnormal screening mammogram revealed a palpable mass above the nipple, associated with skin and nipple retraction. The CC x-ray mammogram (Fig. 4a) shows large architectural distortion central in the right breast, and nipple retraction. The abnormal area measuring more than 5 cm is highly suggestive for the presence of a large malignancy. The US investigation (Fig. 4b) reveals a large, irregularly shaped hypoechoic mass (at least 5 cm) with hyperechoic border which is partly invading into the subcutis. Further, widened ducts are observed, indicating the presence of *in situ* components. The lesion was designated BI-RADS 5 (highly suspicious for malignancy) in both modalities.

Figure 4c shows the photoacoustic data as a MIP in CC direction, and as a direct volume render in Fig. 4d. The lesion cannot be assigned to one confined high intensity area, being mainly composed of several moderate intensity regions of which the largest has a vessel or duct-like elongated shape. Here the contrast of the ROI (*C*) carrying intensities compared with background was estimated to be 3.6. Although difficult to truly link the PA appearance of the lesion to presentations in conventional images, it can be seen that the area is markedly different from the surrounding presumably healthy tissue. This appearance was defined as ‘non-mass’ or ‘scattered abnormality’, the longest axis of the region measuring 41 mm on the MIP.

**Figure 5:**
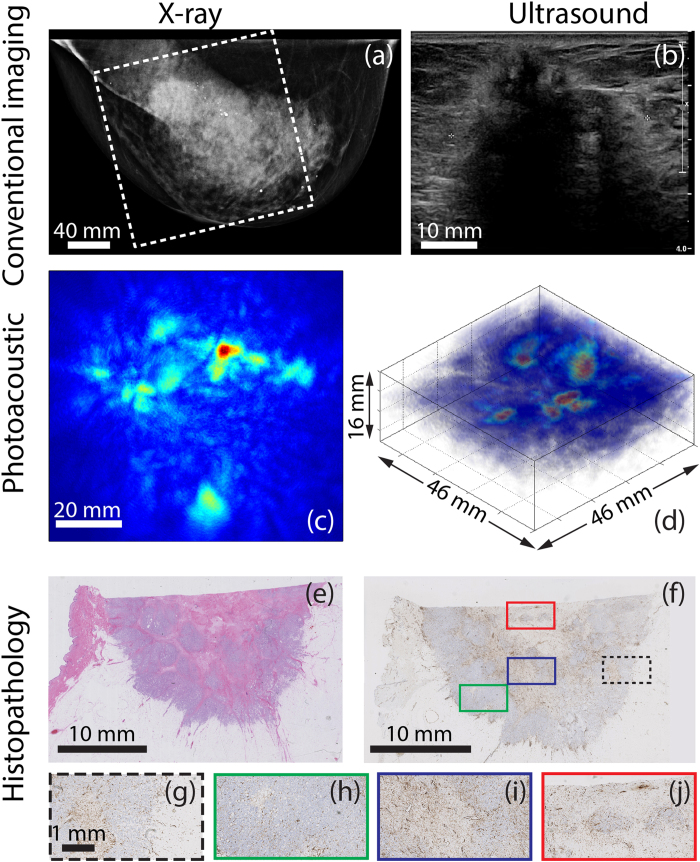
Example of photoacoustic (atypical) ring-like appearance in a 61 year old (P37, Case 4) patient having IDC. **(a)** CC x-ray image of right breast shows high density and highly suspect. **(b)** US image is highly suspect with hypoechoic and irregularly shaped mass, with hyperechoic border and invasion into the skin. **(c)** The PA MIP image highlights the nipple and further shows a regions of high intensity around the lesion. **(d)** The ring appearance is more prominent in a 3D representation of the lesion. **(e)** H&E stained slide show 28 mm grade 2 IDC where tumor cells are mainly present in solid fields at the lesion border in a ring form surrounding the more fibrous core. **(f)** CD31 stained tumor slide shows less vascularity at lesion border, with staining inside the lesion. **(g)–(i) Details of corresponding boxes** of CD31 section show that the density of microvascularity is most pronounced in the area in between the tumor nests which are located between the center and the border of the malignancy.

Histopathological investigation with H&E staining revealed a 63 mm grade 3 IDC, associated with *in situ* components inside the lesion. The malignancy appeared to grow in a pre-existential duct with some nodular fields. The abnormal cells were mainly organized in non-cohesive dissociative infiltrating fields within an area of dense sclerotic fibrous tissue. Owing to the large size of the lesion, a full view of the malignancy cannot be seen in the central H&E section (Fig. 4e); multiple sections spread over the malignancy needed to be investigated for diagnosis. Moreover the extended histopathology protocol was performed retrospectively, after slicing in sagittal direction. Therefore a one-to-one comparison between histopathology and the PA results cannot be made. Figure 4f is the CD31 section with details of the boxed regions in Fig. 5e–i. The overall rather poor amount of microvascularity is mainly confined to the cell-rich fields, and is less prominent but still present in the sclerotic stroma (see also Fig. 4g, for some detail). This overall description generally matches the lesion appearance in PA images, where several non-organized moderate to high intensity regions are observed rather than one confined high contrast area.

### Case 4 (P 37) – Atypical ring appearance for IDC

The 61 year old Caucasian patient presented with a palpable mass in her right breast causing skin deformation. The lesion appeared as a highly dense region in the CC x-ray mammogram (Fig. 5a). In US images (Fig. 5b) the lesion presented as an irregularly shaped, hypoechoic mass with hyperechoic border, invading into the skin. The x-ray and US findings make the lesion highly suspicious for malignancy. In the PA images (Fig. 5c,d), the presence of the nipple^24^ can be observed. Further, multiple moderate to high intensity scattered regions are observed, which appear at the border of the malignancy after co-location with x-ray mammography (see box in Fig. 5a, for PA FOV). The appearance can neither be fully described as ring appearance nor as non-mass appearance, but seems to belong to an intermediate category. The presence of the high intensity foci somewhat organized in a band, led to the PA description of atypical ring appearance. The contrast (*C*) of the lesion is estimated to be 2.2.

H&E staining of the tissue specimen post-surgery (Fig. 5e), reveals the presence of a grade 2 IDC, also showing differences between border and center areas: the tumor cells mainly organized in solid fields at the lesion border surrounding a fibrous core. This matches with the H&E appearance of Case 2 (ring appearance), however in this case vascularity (CD31 stained slides in Fig. 5f and Fig. 5g–j) appears to be less pronounced inside the cell-rich border fields and most pronounced in the areas in between such fields. Thus, while generally the vascularity is not confined to the tumor border as in Case 2, there appears to be a band of higher vascular density inner to the border.

## Discussion

Photoacoustic imaging has been shown earlier to be able to visualize malignancies with high imaging contrast^23^,^24^,^26^,^27^. In this study, we conducted investigations in a small patient population into PA appearances of infiltrating ductal carcinomas (IDC). A first detailed comparison was made of image features in PA with those in MR, and further with vascular staining in histopathology.

Three main types of PA lesion appearances were observed: ‘mass’, ‘non-mass’ and ‘ring’. Further, we observed:

- excellent correspondence in lesion localization between PA and MR images, and a good to reasonably good correlation in lesion shape and appearance,
- good correlation in lesion shape and appearance between PA images and standard histopathology,
- good correlation in certain cases between PA lesion appearance and vascularity patterns from CD 31 immunohistochemical staining pattern .

Here we discuss various points arising from these findings relating to the performance and potential of photoacoustic mammography.

### Photoacoustic lesion appearance

The PA presentations of IDC were mainly ‘mass or confined’, ‘non-mass or scattered’ and ‘ring-like’. These lesion appearances, though not completely clear-cut in categorization, are somewhat reminiscent of enhancement types described in the MR literature namely mass enhancement, non-mass enhancement, focal enhancement, and mass enhancement with ring enhancement^30^.

For the PA appearances, it was considered that hemoglobin (Hb) and oxy-hemoglobin (HbO) are responsible for PA contrast at 1064 nm. There are additional chromophores associated with tumors and normal breast tissue, which could contribute to PA contrast (See further Additional Remarks). However, Hb and HbO are the dominant contrast sources at this wavelength, and thus high intensities in images depict the presence of tumor vasculature, while absorbers such as lipids, and collagen would only modulate the image features^31^. This is supported by the strong correspondence between PA and MR images; and the correspondence between appearances in PA images and CD31 stained tumor slides in 5 out of the *n* = 6 cases.

### Comparison between PA and MR images (*n *= 11 cases)

Generally, excellent correspondence with respect to lesion location was observed, and to a lesser extent with regard to lesion shape and appearance.

Regarding similarities in lesion appearance, we discuss here an exception which is important for understanding the imaging results in the context of tumor vascularization. Case 1 (P38) showed some ring enhancement in MRI, but a mass-like appearance was observed in the PA images. The ring feature was not observed in histopathology, where a more heterogeneous distribution of vascularity is seen (Fig. 2e). For example, some regions of the CD31 stained slide at the tumor margin (Fig. 2g) show higher vascularity, while others also at the margin do not (Fig. 2f). Similarly, the central region shows areas with higher and lower staining (Fig. 2h–i and corresponding colored boxes in Fig. 2e).

Ring enhancement in MR associated with high vascular density at the periphery is predominantly visible early after contrast injection^32^,^33^. In Case 1 however, the ring enhancement is most pronounced at later times, beyond 4.5 minutes post-contrast injection. This is a late phase event when the microvessels start to lose contrast agent. At this stage, a ring appearance is expected^34^ if there is relatively slower outflow from the lesions’ peripheral vasculature compared to that from the inner microvessels; accumulation at the periphery leads to the ring. Hindrance to outflow is usually attributed to inflammation and/or fibrosis outside the malignancy^34^, and the strong desmoplastic fibrotic stromal reaction observed in Case 1’s histopathology makes this the plausible explanation for the ring appearance rather than a high peripheral vascular density. In this situation, in the present embodiment of PAM, where dynamic real-time imaging is not possible, no PA ring-appearance is expected.

It must however be said that the vascular heterogeneity observed in the vascular stained histopathology is somewhat visible in the MR images, but not in the PA images. The reason for this is that the relatively poor resolution achievable with PAM (3.5 mm in axial and transverse directions) does not permit the heterogeneity to be resolved, leading to a more smeared out mass-like presentation.

Lesions that exhibited non-mass PA appearance (*n* = 3), were all estimated to be sized greater than 40 mm in conventional imaging. (Fig. 1e,f and Table 1) In these cases, the lesions are too large relative to the FOV for an accurate PA reconstruction of the lesion and its surroundings, which makes a good comparison in appearance between MR and PA images difficult.

### Comparison between PA images and CD31 stained histopathology slides (*n *= 6 cases)

In all *n* = 28 cases, lesion appearance, shape and size compared well between PA images and H&E histopathology. Regarding the CD31 stained slides, which enabled a comparison between PA images and the vascular distribution, the following could be said:

- In all *n* = 3 cases, the PA mass appearance could be linked to the CD31 (vascular) staining pattern (Fig. 2e–i for example) which was spread over the entire malignancy.
- In the *n* = 1 case, the PA non-mass appearance showed correspondence to the scattered distribution of CD31 stain (Fig. 4f–i) which matched the non-cohesive organization of tumor cells in the lesion.
- In *n* = 2 cases of PA ring appearance, the H&E stains showed a region of tumor cells at the border of the malignancy, (Fig. 2e and Fig. 5e) surrounding a fibrotic core with a lower proportion of tumor cells compared to collagen fibers and fibroblasts. However, only in 1 of the 2 cases of PA ring appearance, was the CD31 staining stronger at the periphery than at the central part of the tumor; the 2^nd^ case showed vascularity to be somewhat confined to a band not at the periphery but well within the malignancy.

We discuss the last case (Case 4: P37) in some detail here. There is a clear presence of a ring in the H&E slide (Fig. 5e) where the tumor cell-rich periphery surrounds a fibrous core. However, in Fig. 5f, CD31 staining is absent in the periphery but is present well within the lesion areas strongly suggesting that the outer cell-rich ring of the H&E slide is poorly vascularized. Figure 5f, with detail in Fig. 5i, shows a number of CD31 stained structures in the fibrous core, which do not seem to be organized into functional vessels. It is plausible that the new vessels created are dysfunctional, and higher amount of CD31 staining in the fibrous core does not necessarily translate to higher hemoglobin concentrations. The end result (Fig. 5f), is a band present well inside the lesion. It is then likely that the PA images visualize this band, which also explains the atypical ring appearance, where the feature is not as clear cut as in Case 2, but with some organization of the scattered PA hotspots in a ring or band. However, if the PA image shows the inner band of functional vascularization, rather than the outer band of dysfunctional vascularization, the lesion size estimated from the PA images would underestimate the histopathological size, while this is not the case and the PA estimated size is larger than the true, histopathologically defined lesion size.

### Additional remarks

Generally, although the CD31 staining is known to be correlated to the MVD and microvessel distribution^35^, it is not always recognized as the most reliable vascular staining^36^. The CD31 staining was chosen based on the pronounced staining compared to CD34 and Factor VIII/von Willebrandt factor in case 1. Therefore, for future research in a larger patient population, a more thorough analysis into the most reliable vascular staining should be performed. Furthermore, not all lesions could be fully processed according to protocol, which made that some malignancies were sliced in sagittal rather than transversal direction.

The Twente Photoacoustic Mammoscope (PAM) images a FOV of 85 × 90 mm, which while large is nevertheless a ROI of the breast. The planar configuration also describes a limited view situation which will hamper the visualization of individual vessels oriented at high angles^37^ to the detector plane. Such vessels of sufficiently large size may indeed be present as feeding vessels to the tumor, however the optical absorption contrast of a tumor originates from the high density of microvessels within the tumor. These are sub-resolution blood vessels whose orientation is randomized within the tumor. The detector with its resolution of roughly 3.5 mm thus is able to visualize the heterogeneous ensembles of the microvessels within the tumor. We believe that the image patterns observed are thus largely accurate to the gross patterns of vascularity in the tumor.

However due to the forward mode of illumination-detection, the imaging depth is restricted by the limited penetration depth of light in tissue, and the entire breast cannot be covered. Therefore, primarily patients with lesions in the cranial side of the breast (when compressed) were included in the study. The reconstructed PA image of the lesion lacks detail in ‘z’ (depth) direction, which makes a one-to-one comparison between (sagitally sliced) histopathology slides, MRI and PA imaging difficult. This study attributes the general PA imaging patterns to various patterns of vascularity. These first insights should be further elucidated using a PA system that can image the entire breast using multiple projections,^50^,^51^ to allow for more detailed depiction of tumor vasculature.

As mentioned earlier, in addition to Hb and HbO there are additional NIR chromophores associated with tumors and normal breast tissue, which could contribute to the PA contrast. Water content, especially in the form of free (unbound) water, is significantly higher in malignant tissue compared to surrounding healthy glandular or fatty tissue from optical spectroscopy and Magnetic Resonance Spectroscopy (MRS) studies^31^,^38^,^39^,^40^. Lipid content is also generally thought to be decreased in tumor tissue compared to the surrounding^31^,^38^ while necrotic cores can be lipid rich instead^40^. Collagen concentrations can also vary within a tumor with fibrous stroma, as seen in specific cases presented here. It is especially the water content, which, based on its relative absorption coefficient at 1064 nm and its average concentration in healthy and malignant breast tissue is expected to influence the PA imaging results at this wavelength. The low absorption coefficient of collagen at 1064 nm and the relatively small difference in fat content between healthy and diseased breasts are believed to play a minor role.^30^,^31^

While this contrast is favorable for future application in detection and diagnosis settings, it could however make the interpretation of images made with a single wavelength ambiguous. Specificity using a single wavelength is also expected to be limited because of the difficulty in discriminating the contribution of hemoglobin from that of sometimes heterogeneously present water, fat and collagen to the PA appearance. To ascertain the relative contribution of these chromophores to PA lesion appearance and contrast, multi-wavelength PA measurements are necessary.

The problem in making accurate estimations of the chromophore concentrations, is that PA images are not proportional to their absorption coefficients^41^. This is due to the presence of spatially distributed and wavelength-dependent attenuation, which leads to unknown fluence distributions. Further, spatial variations in the Grüneisen coefficient, which relates the absorbed light to PA pressure generation, are also expected to confound the quantitation of chromophore concentration. While much work is being done in the area of quantitative PA imaging^41^, accurate estimations in the breast with the vast amounts of tissue present is challenging. However, much can be learnt even qualitatively using a few selected wavelengths^42^. For example 755 and 1064 nm are interesting wavelengths due to the opposing behaviors of Hb and HbO absorptions with the former having higher absorption at 755 nm compared to at 1064 nm, and vice versa with HbO^42^. Further, water and fat absorptions are near negligible at the shorter wavelength compared to at 1064 nm.

## Conclusions

Overall this work shows that PA breast imaging has potential in visualization of cancer. For the first time, we observe patterns in PA image intensities which while not being completely clear-cut in categorization, can be described as mass-like, non-mass and ring appearances. A comparison of the image features in PA with those in contrast enhanced MR, and with vascular stained histopathology, leads us to attribute PA intensities to the presence of vascularity. The contribution of water and fat to the PA intensities at 1064 nm is probably lower, but will have to be confirmed by multiple wavelength studies. The elucidation of PA patterns of malignancy should be pursued in a large patient population. Such knowledge can potentially contribute to the development of PA image descriptors which in the long run may serve as diagnostic criteria for the technique.

## Methods

### Diagnostic trajectory and patient inclusion

The study protocol and informed consent procedure were approved by the Medical Ethics Review Board of Medisch Spectrum Twente (MST), and the study was registered in the Dutch Trial Register^43^. All patient measurements were performed at the Center for Breast Care of the MST in accordance with the approved protocol.

Standard diagnosis was based on information integrated from the triple assessment comprising clinical investigation; imaging using x-ray mammography (XRM) and ultrasound (US) echography; and core needle biopsy (CNB). Lesions suspect or highly suspect (4 or 5 on the BI-RADS classification scale^44^) in one or both imaging modalities, as judged by a breast imaging radiologist, are considered for the study. Between April 2012 and April 2013, 29 consenting adult female patients with the above criterion were included.

### Photoacoustic breast imaging measurements

**Figure 6:**
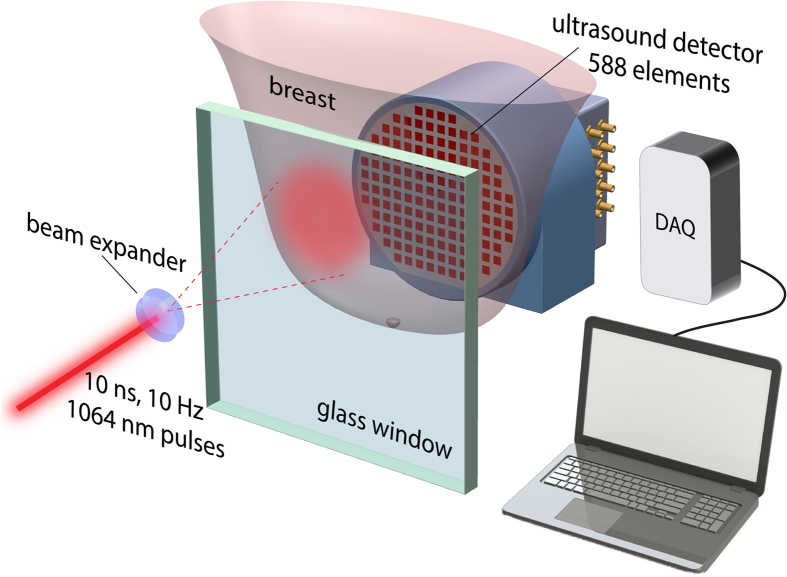
Schematic of the imager of the Twente Photoacoustic Mammoscope: The patient lies prone on the bed (not shown), with her breast through an aperture. In the imager, the breast is immobilized between a glass window and an ultrasound detector matrix. Light from a Q-switched Nd-YAG laser at 1064 nm (10 ns pulses, 10 Hz) expanded to a diameter of approximately 70 mm illuminates the breast through the window. The 588 detector elements with a central frequency of 1 MHz, are arranged in a diameter of roughly 85 mm. Analog front-end electronics are mounted close to the detection elements, and the data is digitized and read into the computer for off-line image reconstruction^47^.

PA imaging took place after conventional imaging, but prior to CNB to exclude the likelihood of imaging hematoma post-biopsy. A technical description of the imager, the Twente Photoacoustic Mammoscope (PAM), and the imaging protocol are concisely presented here; further details can be found in Refs 23, 24, 25 and 45. During the PA investigation, the patient lay prone on the bed with her breast through the aperture. The breast was immobilized under mild compression in a craniocaudal (CC) direction between a glass window for laser illumination and a 2D ultrasound detector array. (Fig. 6) The relative position of the compressed breast with respect to the detector array was recorded by making a photograph through the glass window in CC direction. This information was used to overlay PA images on x-ray and MR images (see further).

Pulsed light (10 ns, 10 Hz) at 1064 nm, from a Q-switched Nd:YAG laser (Continuum Surelite, California, USA) was used for excitation. The beam was maintained at a fixed position on the breast surface, with a beam area of approximately 35 cm^2^ and an energy of 350 mJ per pulse, giving a radiant exposure of 10 mJcm^−2^. This is well below the Maximum Permissible Exposure (MPE = 100 mJcm^−2^) on the skin for the parameters of the laser light used^46^. In cases when the detector array was not completely covered by the breast, the uncovered portions of the detector were shielded from direct light to protect the sensitive PVDF elements.

The 2D ultrasound detector array consists of 588 elements in an approximately circular layout with diameter of 85 mm^25^. The PVDF elements possess a central frequency of 1 MHz with a bandwidth of 130%. The elements are grouped into 10 sectors; each sector’s approximately 60 elements serviced by an Application Specific IC (ASIC), which buffers and amplifies the signals. Each ASIC has a single output which leads to a 10 input summing amplifier. In the upgraded PAM, the outputs of the 10 ASICs are made to bypass the buffers, amplifiers and summing amplifier. An independent interface unit was developed with 10 channel buffer-amplification based on the AD797ar ultra-low noise op amp. The 10 outputs are the acquired at a time using two 8- channel digitizers (National Instruments, NI PXI 5105, 60 MS/s, 12-bit) controlled by a Labview program (National Instruments). With averages over 10 signals per acquisition, an imaging time of 10 minutes was required for the complete detector area covering a FOV of 90 × 85 mm^2^ on the breast.

Signal processing, image reconstruction and analysis were performed using Matlab (R2011b, the Mathworks Company). Prior to reconstruction, the high signal from the breast surface was removed by zero padding, so that signals from the PA volume occupy the available dynamic range. Signals were filtered with a band-pass Butterworth filter (cut-off frequencies 0.2 MHz and 1.7 MHz) to remove the low frequency trend and high frequency noise. Reconstruction was performed using an acoustic backprojection algorithm, assuming a homogenous speed of sound (SOS) of 1540 m/s. The inaccuracies arising from the use of a homogeneous SOS are estimated to be minor with respect to the system’s resolution (approximately 3.5 mm in both axial and lateral directions)^25^,^44^. Sagittal slices of the reconstructed volume were Hilbert transformed, and all voxel intensity values were normalized to the maximum intensity in the volume and scaled to range between 0 and 255.

The reconstructed PA volume was examined for the presence of high-intensity areas. The relative position of the breast and detector array from the photograph allowed the location of the PA volume in the breast to be ascertained. The orientation and magnification of the breast contour from the photograph were adjusted to match the CC x-ray image and the MR image, so that the maximum intensity projection (MIP) of the reconstructed PA volume can be overlaid on either image at the correct coordinates. This permitted comparison of PA image features with lesion features in the x-ray and MR images. In case of good co-location of the PA intensity distribution with the lesion in x-ray, the PA feature was judged to be originating from the abnormality, and is referred to as a PA lesion. However, since the amount of compression varied from heavily (XRM) via slight (PAM) to none (MRI), the tissue was deformed differently during all investigations and one-to-one comparisons cannot be made. The lesions were classified according to their appearance on the MIP (see further). The size of the PA lesion is estimated via a manually drawn contour around the lesion on the MIP using information from conventional imaging.

### Estimation of PA lesion contrast (*C*)

When high intensity PA pixels were organized similar to lesion appearances from experiences in conventional imaging modalities, these structures were referred to as PA lesions. A sub-volume was defined include the depth (cranio-caudal) extension of the lesion. A contour was manually drawn around the lesion on the maximum intensity projection (MIP) of the sub-volume. This contour was used to measure the maximum dimension of the lesion in the CC projection and to calculate the average intensity inside the lesion. Ten random areas outside but close to the lesion were then chosen. For each area, the average intensity was calculated in order to be able to calculate the contrast of the lesion (lesion intensity divided by background intensity). To reduce subjectivity in the choice of the specific background area, the average of the ten calculated contrasts was used to obtain *C*.

### Magnetic Resonance Imaging (MRI) protocol and analysis

A dynamic contrast enhanced MRI investigation after triple assessment is recommended in case of uncertain findings in x-ray due to high breast density, conflicting findings between x-ray and US, inconclusive diagnosis or the requirement for assessment of lesion extent for surgery planning. A 1.5 or 3 Tesla MR system in combination with a dedicated 7 channel Sense Breast Coil (Philips Medical Systems, Best, The Netherlands) was used. High-resolution, anatomic T2weighted images, and dynamic T1 weighted images before and after gadolinium injection were acquired. Acquisition was usually 1 pre- and 3 to 4 post-contrast images over a maximum duration of 9 minutes after contrast injection. In pre-menopausal patients, MR imaging was scheduled in the 2nd week of the menstrual cycle when the enhancement of the glandular tissue is known to be least pronounced^47^.

The images were interpreted by a specialized breast radiologist with extensive MR experience. The T2 weighted images were examined for morphological changes and architectural distortion of breast tissue. Following subtraction of pre-contrast images, the T1 weighted post-contrast images were examined for abnormally enhancing areas and the dynamic enhancement curve were studied. The hemodynamic characteristics of a malignancy cause rapid enhancement followed by a washout phase^12^. The radiologist usually described the lesions as abnormally enhancing areas with or without evident mass contribution (space occupation and distortion). Further information concerns the shape, the borders, the heterogeneity, the presence of ring enhancement, the presence of spiculae and the presence of other foci of the lesion elsewhere in the breasts.

PA and MR images were compared for the presence, size and appearance of abnormal areas. The pendant position of the breast during MRI made it possible to retrospectively compare lesion positions in all images with the information obtained from the digital photograph.

### Histopathological assessment

Excised tissue was examined at the Laboratory for Pathology East Netherlands (LabPON). The orientation of the breast or lump was marked by the surgeon, after which it was fixated in formalin 4% for at least 24 h and cut in approximately 5 mm thick sagittal slices. For further investigation, a specimen radiography was made from the sliced tumor, and supported by this reprensentative blocks from the tumor and its surroundings were taken and embedded in paraffin blocks. From these, 2 *μ*m slides were cut and stained with Hematoxylin and Eosin (H&E). These slides were reviewed by a pathologist who reported on the status of the surgical resection margins; on histologic type (according to the WHO classification) and histologic grade (according to the Bloom-Richardson scale^48^); on hormone receptor status (ER, PR and HER2/neu); presence of *in situ* components; and on complete size of the invasive (and/or non-invasive) part of the lesion.

In a subset of patients in which the lesion in PA images could be related with certainty to the abnormality visible in conventional imaging, an extended histopathology protocol was performed. The breast or lump was sliced in transversal direction, i.e. to obtain slices in the direction from which most information is available from the PA images as a consequence of the forward mode imaging. From the tumor central slices were embedded for paraffin in whole mount blocks. In addition to the conventional stains, one or more slides of the malignancies were stained for vascularity using anti-CD31 (referred to as CD31 in this manuscript) and visualized with DAB (diaminobenzidine) antistaining which gives a brownish color to the antibody. This antibody is mainly located at the borders between endothelial cells and gives information about vascularity^49^. If possible, this CD31 stained histopathology slide contained a complete cross-section of the malignancy. However, if the lesion was too big for this, and whole-mount (4 × 4 cm^2^) sections could not be obtained, multiple slides were cut and stained for vascularity. These slides together either composed an entire cross-section of the tumor or represented areas spread over the entire lesion, including center and border areas. The CD31 stained slides and the H&E stained slides were digitized using the Hamamatsu Nanozoomer 2.0-HT slide scanner (Hamamatsu, Japan). The pattern and amount of microvascularity as well as the size, shape, type and grade of the lesion were qualitatively compared to the appearance patterns on PAM and MRI.

## Additional Information

**How to cite this article**: Heijblom, M. *et al.* Photoacoustic image patterns of breast carcinoma and comparisons with Magnetic Resonance Imaging and vascular stained histopathology. *Sci. Rep.* **5**, 11778; doi: 10.1038/srep11778 (2015).

## Figures and Tables

**Table 1:** Photoacoustic and MR representation of the identified lesions for the 11 patients for which MR images were available.

P	PA-MR imaging co-localization	PA lesion description	MRI lesion description	PA size (mm)	MRI size (mm)	Lesion size (mm)#
*Lesion size* *<* *40* *mm*#						
38	Good	Heterogeneous *mass*	*Mass* with ring enhancement; enhancement most pronounced at border, surrounding several hypodense fields.	19	22	19
55	Good	Irregularly shaped *mass*	Irregularly shaped *mass*	28	50	34
56	Poor-reasonable	Spherical *mass*	Spherical *mass*	11	32	22
58	Reasonable-good	Star-shaped *mass*	Star-shaped *mass*	19	55	22
61	Good	Irregularly shaped *mass*	Irregularly shaped *mass*	21	20	16
62	Good	Spherical *mass*	Spherical *mass*	16	19	18
67	Reasonable-good	Oval *mass*	Oval *mass*	16	15	13
70	Good	Lobed *mass*	Oval *mass*	21	30	19
*Lesion size* *>* *40* *mm*#						
40*	Reasonable	*Non-mass*; area with several unevenly distributed and sized foci	Diffuse non-mass with *mass* component in lateral upper quadrant; architectural distortion with malignant enhancement	45	N.A.	>60 mm*
49	Good	*Non-mass*; area with multiple enhancing foci	Non-mass, with possible *mass* contribution	54	50	40
52	Good	*Non-mass*; area with several unevenly distributed and sized foci	Strongly heterogeneous *mass,* vascularity most pronounced at border	53	55	60

^#^The gold standard for the lesion size is the histopathologically measured size, in case no results from histopathology were present, the size on x-ray imaging is taken as gold standard.

^*^In this patient the size of the lesion could not be assessed, because it occupied a large part of the entire breast and was largely associated with diffuse non-mass appearance in MRI.

**Table 2:** Comparison between PA lesion appearances and CD31 stained (vasculature stained) histopathology for the 6 patients for which the extended pathology protocol was being performed.

Case*	P	Type of malignancy	PA lesion description	Extended histopathology description	PA size (mm)	pathology size (mm)
1	38	IDC; Grade 3	*Mass*	Vascularity heterogeneously spread, but higher density at border	19	19
	55	IDC; Grade 2	*Mass*	Vascularity spread	28	34
	70	IDC; Grade 3	*Mass*	Vascularity heterogeneously spread, but higher density at border	21	19
2	47	IDC; Grade 2	*Ring*	Vascularity confined to border	24	24
3	39	IDC; Grade 3	*Non-mass*	Vascularity most pronounced in non-cohesively organized fields	41	63
4	37	IDC; Grade 2	Atypical *ring*	Vascularity heterogeneously spread	31	28

^*^Specific case numbers in this article; IDC = Infiltrating Ductal Carcinoma.
